# Ambiguity in knowledge transfer: The role of theory-practice gap

**Published:** 2010

**Authors:** Mohammad Ali Cheraghi, Mahvash Salsali, Mahmoud Safari

**Affiliations:** * Assistant Professor, School of Nursing and Midwifery, Tehran University of Medical Sciences, Tehran, Iran; ** Associate Professor, Department of Post Graduate, School of Nursing and Midwifery, Tehran University of Medical Sciences, Tehran, Iran; *** Tenure Faculty Member, School of Nursing and Midwifery, Hamadan University of Medical Sciences, Hamadan, Iran

**Keywords:** Iran, nursing, qualitative research, nursing theory, knowledge

## Abstract

**BACKGROUND:**

In spite of much literature written about the theory-practice gap in the international nursing journals, there is evidence that indicates this subject has not been probed comprehensively since nursing education was transferred to universities in Iran. In the recent years, the public and the government have criticized Iranian nurses because of poor quality of patient care. Although this subject has been lamented by some researchers, there is no comprehensive work on how this gap resulted. In the process of a larger study on “nursing knowledge translation to practice”, of one PhD thesis, this process was explored.

**METHODS:**

Using grounded theory analysis, indepth interviews were undertaken with a purposive sample of 29 nurses, with different levels of experience, from the school of nursing in Tehran University of Medical Sciences in 2006 from January to August. Data were analyzed using the constant comparative method.

**RESULTS:**

Three main themes emerging from this study included clinical behavior structure, paradoxical knowledge and practice, and divergent nursing organization.

**CONCLUSIONS:**

It seems that nursing education with some praxis and paradoxes in the realm of nursing knowledge and practice, along with divergent organizational structure have decreased nurses’ ability in applying their professional knowledge and skills in order to bridge the gap between theory and practice. Moreover, in spite of increased academic input into nursing education, clinical behaviors of both education and practice settings was perceived as “traditional routine-based”.

Nursing in the 21^st^ century involves complex and highly specialized care. To meet the demands of health service provision, nurses need to have sound theoretical knowledge as well as proficient practical skills.^1^ Research in several countries provides consistent evidence of the existence of a theory- practice gap in nursing. Nursing education in Iran has undergone much change since the first training schools for nurses were opened in the 1940s. Having become accepted as a respectable occupation within Iran, nursing has now turned its attention to the academic paradigm. At present, the nursing programs in Iran offer a four year baccalaureate as a community oriented nursing program with the philosophy of health for all in nursing. The four-year nursing education program consists of three years of theoretical education in which courses are pursued in schools of nursing and one year of clinical practice in hospitals.^2^ Moreover there are 184 centers offering the BSc degree, 18 centers offering the MSc degree, and 11 offering the PhD degree in nursing. Furthermore, there is a centralized curricular in nursing education at all these nursing programs in the country, and all nurses receive the same type of education.^3^,^4^

Aims of nursing education principally center on the transmission of nursing knowledge, and assisting nursing students to acquire the necessary skills and attitudes associated with nursing practice.^5^ With the transition of nurse education into universities, concern has been raised about the usefulness of theoretical knowledge in practice.^6^ Studies in this arena were named metaphorically as theory-practice gap. This so called theory-practice gap appears to be a global phenomenon and has been repeatedly debated within nursing; with many pages of journals devoted to the issue.^7^–^14^ Most of these studies have linked the problem to the nurses’ knowledge and skills. In fact, the much written about theory-practice gap indicates that knowledge gained by nurses in the classroom may be perceived to bear little resemblance to what is needed in practice; that is, academics are perceived to teach content that is inappropriate for use in the practice arena.^10^ Unfortunately there is literature to suggest that nurses do not always provide the care they had been taught in the classroom and consequently, they cannot perform competently in clinical settings in their transition period; do not integrate the theory to practice, and do not systematically utilize the research findings in their daily nursing cares.^15^–^17^ Accordingly, in recent years, the public and the government have criticized Iranian nurses because of poor quality of patient care,^18^ and the divergence between nursing theory and clinical practice has been recognized and lamented by some nursing researchers in Iran. According to Nayeri et al,^19^ managers believed that staff’s lack of knowledge is a significant barrier to being productive and providing high quality of care and Hagbaghery et al^20^ implied that the nursing educational system did not function well. Moreover, nursing students were not satisfied with the clinical component of their education. They experienced anxiety as a result of feeling incompetent and had a lack of professional nursing skills and knowledge to take care of various patients in the clinical setting.^21^

Although there are some limited literature on theory-practice gap in Iranian researchers work, nurses’ views and experience on the knowledge translation process have not been studied comprehensively. Thus, an important area for research is to obtain nurses’ perspectives on knowledge translation to practice for effectively bridging the gap. This is mainly essential for administrators and educators to consider when designing strategies to improve practice settings and educational practices. Since exploring the knowledge translation process closely depend on recognition of linkage intensity between theory and practice in two settings of work environment and academic area, this study focused on the experiences, perspectives and perceptions of Iranian nurses about metaphor of theory- practice gap as two sides of a coin: theory- practice/practice-theory gap.

The aim of this study was to explore the experiences, perspectives and perceptions of Iranian nurses about the metaphor of reciprocal theory- practice/practice-theory gap.

## Methods

### 

#### Design

Data were collected and analyzed using a grounded theory approach.^22^ This approach was selected because nurses’ practice takes place in a multidisciplinary team and grounded theory focuses on identification, description, and explanation of interactional processes between and among individuals or groups within a given social context.^23^ In addition, Struauss’s version of grounded theory emphasized meaning, action, and process, consistent with his roots in pragmatism and symbolic interactionism.^24^ Taped in-depth interviews and participant observations were conducted.

#### Participants

Purposeful sampling was used at first and continued with theoretical sampling according to the codes and categories as they emerged. Participants initially comprised of 21 internship nursing students at their last semester, junior nurses (newly educated registered nurses called “Tarhi” in Iranian terminology), experienced staff nurses, supervisors, managers, and lecturers from two large hospitals covered by the Tehran University of Medical Sciences and School of Nursing and Midwifery in Tehran, Iran. As well, according to the need for saturation in some categories in the theoretical sampling stage, interview with two nursing managers, three lecturers, one medical doctor, and two hospitalized patients were conducted. The managers were representatives of service and educational top decision-makers. Sampling commenced in the School of Nursing and Midwifery with internship nursing students, and then was extended to the hospitals.

#### Interviews

The institution’s internal review board approved the study before data collection began. Confidentiality between participants and the author was assured by acknowledging the confidential nature of the data and the right to withdraw at any time. Data collected through in-depth, semi structured interviews. Based on the participants’ request, interviews were carried out in the School of Nursing and Midwifery, hospitals, managers’ offices in the university, and in the Ministry of Health and Medical Education.

At the beginning of each interview, participants were asked to describe one of their clinical placements, own working shifts and then to explain their own experiences, perceptions, and perspectives about what “knowledge translation means and the linkage of theory and practice”. For example, they were asked: “In your opinion, what factors facilitate or inhibit effective convergence of theory and practice in nursing?”

The interviews were tape recorded, transcribed verbatim and analyzed consecutively. All interviews took one session according to participants’ requests, except two cases that included two sessions. Each session lasted from 45 to 90 minutes. As well, the transcripts with open coding were e-mailed to some of the interviewees such as internship students and lecturers to assure that there were no misunderstandings and to improve the reliability of this research.

#### Observation

Participant observation involved following individual nurses and students around as they were providing care or sitting in the corner of a ward or nursing station, participating in classrooms, nursing clinical laboratory in the school, and observing. The center of attention of participant observation in classrooms was on educational content, teaching-learning approach and activities, and in practice on the nurses’ and internship student nurses transactions and behaviors. Field notes were written to describe the observations and later analyzed, again using the constant comparative method.^25^

#### Data Analysis

Data were analyzed using the constant comparative method.^25^ The process of conducting interviews, transcribing the recordings, and analyzing data occurred simultaneously. In fact, each interview provided direction for the next one. Data collection and analysis continued until saturation; that is, no new insights were gained from further interviews. Open, axial and selective coding was applied to data.^23^ Codes and categories from each interview were compared with codes and categories from other interviews for common relationships. Axial coding was concentrated on the conditions and situations which cause a phenomenon to take place and the strategies applied to control the phenomenon. This process allowed links to be made between categories to their subcategories and then selective coding developed the main categories and their interrelations.

Credibility was established through participants’ revisions using member checking, prolonged engagement with participants and peer checking. Maximum variation of sampling also confirmed the conformability and credibility of data.^26^ As a further validity check, two expert supervisors and two other faculty members did peer checking on some transcripts. Results were also checked with some of the nurses who did not participate in the research and they confirmed the fitness of the results as well.

#### Ethical Considerations

Ethical issues in this study involved the assurance of confidentiality and autonomy for the participants. The Internal Review Board of Tehran University of Medical Sciences, School of Nursing and Midwifery, approved the study before data collection began. Permission was sought from the participants for audiotaping interviews and the hospital directors, head nurses, and vice dean for education in the school of nursing and midwifery had also agreed to participant observation.

## Results

The results presented here include a report of some main themes identified through the data analysis of a grounded theory study. Accordingly, the interrelationship between core variable and other themes has not been discussed here. As well, individual participants’ characteristics are presented in table 1.

**Table 1:** Individual characteristics of participants

Participants	Age (year)	Sex		Years of service as nurse (year)	Level of education		
		M	F		BS	MSc	PhD
Internship students	22-27	2	7	4^th^ year of education	9	-	-
Junior nurse	24-27	1	2	1-1.5	3	-	-
Staff nurse	37-40	1	1	13-16	2	-	-
Supervisor	44-49	-	2	20-28	2	-	-
Manager	40-70	-	4	17-52	1	2	1
Educator	36-50	3	3	8-25	-	6	-
Physician	41	-	1	13	-	-	1
Patient	34-58	1	1	-	-	-	-

Three main themes emerged from the data including “clinical behavior structure”, “para-doxical knowledge and practice”, and “divergence in nursing organization” and their subcategories are presented in table 2, and with more details at below:

**Table 2:** Emerged themes and subcategories

Themes	Subcategories
Clinical behavior structure	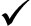 Traditional routine-based delivery nursing care
	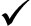 Traditional-based clinical education

	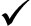 Educational program structure
	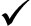 Educational content inadequacy
Paradoxical knowledge and practice	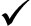 Clinical educator competency
	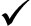 Unstructured evaluation
	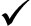 Formless theoretical teaching-learning process

Divergence in nursing organization	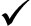 Content dimensions: environment/culture
	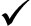 Structural diminutions: hierarchy of authority/standard/expertise condition

### 

#### Clinical Behavior Structure

“Clinical behavior structure” was one of the main and broad themes that were categorized as following:

#### Traditional Routine-Based Delivery of Nursing Care

The dominant clinical behavior among the practice-based as well as educational nurses was traditional. One of the junior nurses described her experience as: where is the nursing knowledge? You are just a simple worker with some routine-bound duties; such as, giving the drugs, injections, change the sheets, and wound dressings; the duties that “Behiars” (meaning practical nursing assistants in Iranian term) are doing; 6-month training to do this type of nursing is enough.”

One of the main intervening conditions on this regard has been related to senior-centered behaviors. As one junior nurse indicated: “in this hospital, nurses were educated within the old hospital-based program, are dominant; you see many nursing procedures done in the wrong way, different from what we are taught in the clinical lab in the school. If you question this, they would chastise you as a challenging staff member; frequently they say we do not do that in this way on this ward.”

On the other side, the above mentioned status continues in challenging condition as one of these senior nurses mentioned that: “I do not know, what they [meaning university educators] are teaching to students; these juniors even do not know how to take care of an IV line, as their teachers.”

Moreover, contextual data revealed that inappropriateness in some staffing ratios, such as nurse/patient ratio, heavy workload, and nonnursing duties are some causal and intervening inhibitors against advanced practice nursing that was mentioned repeatedly. A general practitioner who participated claimed: “the ratio of doctors and nurses to patients is very inappropriate, in this ward, you can see, we have 2 nurses for 32 patients in this evening shift in which they would only be able to give their drugs and do our orders.” Furthermore, these concerns were affirmed by patients. A patient stated: “nurses are doing whatever the doctors say, they give our drugs, monitor our blood pressure, pulse, and temperature; take blood samples, change our IV lines; God bless all of them.”

Based on contextual data, one of the common recent ways of deconstructing the routine approaches was continuing education for staff which the majority of the participants found ineffective. One experienced staff nurse said “although, we had a workshop on documentation, you can see the so-called nurses’ notes in the chart; well, patient’s general condition isn’t bad; drug orders were done; vital signs were checked; blood sample should be taken for FBS tomorrow.”

#### Traditional-Based Clinical Education

The clinical teaching climate was found not to be conductive and fruitful to the students’ clinical learning in order to integrate theory and practice. Students lacked qualified teachers for guidance and supervision, and experienced an autocratic relationship with them. One internship student said: “our clinical experience was all about obeying the staff nurses to do their duties, division of the patients among students; you have to do their routine works.”

Field notes and memos revealed that the dominant process and interactions in the clinical setting were perceived as a non-scientific atmosphere. One of the students said: “we hear from medical students and their teachers, for example, according to those research findings, this way is better; that text says this is better; they have counseling between different specialties; they have morning report, case report; but in our nursing, there is not any scientific and human relationship among nurses; any information is being cited from texts or nursing research almost never.” In brief, this category emphasized the reciprocal theory-practice gap, more specifically, as practice behavior dominated by educators.

#### Paradoxical Knowledge and Practice

Paradoxical knowledge and practice was categorized as following:

#### Educational Program Structure

Twenty-six participants emphasized the significant function of nursing education in the content of developing knowledge in the minds of nurses. One senior nurse manager commented: “our academic education does not prepare students to be competent and skillful. It gives medical-centered theoretical knowledge from western texts to them that are not applicable, and are not focused on our local and regional health needs.” In regard to this paradox, one junior nurse said: “I have heard more about auscultatory gap in blood pressure monitoring in classrooms, but in the clinical education have never seen that, and other instances like this.”

Role models, as a most important intervening condition, also played an interactive significant role in the reluctance of nurses to follow nursing information enthusiastically as participants mentioned. One of the students stated: “my classmates think the best nurses are the nurses who have more medical information. They do not listen to the sections on nursing care during the lessons in classrooms. Essentially, the manner of the teachers exacerbated this issue; they give us an extensive range of disease-related pathophysiological information, but do not spend even 10 minutes on the nursing care in a two-hour class.” These statements were confirmed by participant observation from classrooms.

#### Educational Content Inadequacy

Contextual data indicated that basic sciences such as anatomy, physiology, and pharmacology are required to be more scientific. It seems to be an evidence of the paradox. On the other side, many participants identified the requirement for certain contents to be included within the curriculum such as communication skills, research concepts and principles, rehabilitation, cultural, ethical and spiritual subjects, special courses for clinical nurse specialists, computer and information technology (IT) applications, and efficient practical English courses.

#### Clinical Educator Competency

According to contextual data, memos and field notes, specialty in a particular clinical field, being knowledgeable theoretically and practically, and practical experience alongside being a reference to staff to carry out care, and having respectful relationships with staff, doctors, and students, as some of the most important intervening conditions, were emphasized as characteristics of a clinical educator by participants. However, the contextual data revealed that the majority of participants perceived them as inexpert, unconfident to carry out nursing care, with role conflict between student supervision, staff, doctors, and patient care, which in turn, widened the gap between theory and practice reciprocally.

One of the nurses in the coronary care unit, stated: “she [meaning one of the faculty clinical educators] is very respectful for all; she is very useful to us; at every moment, you can learn a new thing from her; she transfers her experiences, and knowledge to staff; we often participate on her ward round; wishing all of the university educators were alike her.” As well, one of the nursing managers commented that: “they [meaning education executives] prefer to register newly postgraduate nurses without any additional clinical preparations for clinical teaching; whereas, we have qualified nurses that would be able to teach their students clinically.”

Also, lack of postgraduate students’ involving in the practice setting as an intervening facilitator in order to foster competent clinical educators was emphasized. One of the top managers claimed that: “as evidence, how many of the postgraduate students are involved in practice, at least as a part of their education? I can claim, none; our PhD students think that they are not nurses.”

#### Formless Theoretical Teaching-Learning Process

All of the students and junior nurses stated that lectures in the school, as the dominant approach, were not interactive and did not focus on applied knowledge, communication skills, problem-solving, decision-making, and critical thinking. One of the students stated that: “we have not had a lesson on research methods.” As well, one of the qualified lecturers stated: “the lecturers do not know how to use new teaching-learning strategies. Decision-making and critical thinking does not occur simply as a result of knowing about it. Having 10 students for each teacher in clinical training, unmotivated students and educators, shortage of needed facilities, and more important than others, educational management which is awful, not only in nursing but also in other educational systems, are the main intervening conditions in the way of employing these approaches.” Contextual data and field notes included the requirement to change the methods of teaching to encourage critical thinking and problem solving in order to close the gap.

#### Unstructured Evaluation

Contextual data, field note and memos indicated that educators and managers, as evaluative agents in practice settings, had not been able to make an objective evaluation of students and staff. One of the experienced staff nurses stated that: “we have not a competent evaluative system to be able to separate the qualified staff from nonqualified ones; for example, when people are presented as ‘prototype nurses’ you get bewildered; I mean, persons are chosen based on relation not rules; in this way, the qualified and knowledgeable persons are not placed appropriately in positions; criteria are not scientific values.”

Based on contextual data, in the education system that expected to be an initiator in applying the scientific approaches there were problems, too. One of the students explained that: “we do not know how they [meaning educators] evaluate us? You cannot find them in the ward; after allocating patients or ward rooms to us, they disappear and at the end of the shift they come back to check us to be present; rarely some of our good educators define some criteria for evaluation; have you seen the evaluation form of the faculty? This socalled form is for all clinical area with some very subjective and general items, such as the appearance, makeup, clothing and so.” As well, one of the lecturers, while criticizing the evaluation approach, said: “multiple choice questions have been accepted as an existing approach, and you should act like others, otherwise they [meaning colleagues] would be chastised.” Furthermore, absence of any quality control, which in turn, acts as an intervening factor in identifying advanced practice nursing, was reported by the majority of the interviewees. One of the expert nurses with 16 years of service said that: “as we have no standardized structure and system of quality control or quality assurance; which items should be assessed in the hospital, is by chance.”

Final inference from the contextual data, field notes and memos revealed that paradox was the main property in this category, which in turn, is interplay among these subcategories.

#### Divergence Organization of Nursing

Contextual data, field notes and memos indicated that nursing in Iran is organizationally poor in both cultural and structural elements. The alignment of subcategories in this domain was achieved after theoretical sampling from the texts, according to Daft’s classification on organizational structure as follows:

#### Content Dimensions: Environment/Culture

According to the contextual data, filed notes and memos, the nursing organization confronts both stable and turbulent work environments. The traditional routine-based methods of delivery of care have induced a stable environment. With growth in the university, the emphasis on a new brand of nursing, challenges between nursing as a subordinate system and doctor-centered structure, as well as simultaneously between nursing service section and education, also critiques of the qualifications of nurses from this system of education, low salary, public perceptions of the lowstatus of nursing has made the environment much more turbulent as some important intervening and causal conditions. Positive human relations and communication skills, and possessing a sense of responsibility and accountability were emphasized by the participants as an inevitable requisite.

All participants from both settings cited the divergent climate between practice and education. One of the lecturers said that: “practice and education in our nursing are two parallel lines that, in my mind, will never come together.” Also, one of the practice managers stated: “we have not had even one session with the school of nursing; they think they are very knowledgeable and we are not scientific.” As well, the issue of divergence, one of the supervisors said: “in the past year, we had just one session with school of nursing, on our request, which have been on challenge about ‘sheet changing’ by students.”

#### Structural Dimensions: Hierarchy of Authority/Standard/Expertise

Participants stated that in order to get convergence between practice and education, structured changes were required such as different hierarchy of authority with definition of new roles or bilateral responsibilities for academic and practice nurses, clinical educators appointed from practice staff, and reciprocal supervision from education. Shared programs to facilitate knowledge translation and bridging the gap such as collaborative research projects; unity among main policy making sections of nursing such as, board of nursing, nursing office in the Ministry of Health and Medical Education, Iranian Nursing Organization (INO), service related nursing offices in the universities, and nursing schools are needed. One of the top managers said: “this unity would be achieved, if the academic nurses abandon egoism; indeed, try to encourage collegiality among practice and academic nurses is the intellectual way of professional success.”

Specialization and standardization such as clinical nurse specialization, appropriate nurse/patient ratio, reasonable salary, and quality care and education in both settings were emphasized. One of the lecturers said that: “expert practitioners or academic educators who are experienced and familiar with a domain of activity are very effective in bridging the gap you are mentioning.” Other one stated that: “expert nurses are initiators of establishing standards in nursing care delivery; indeed, they are change agents that our nursing needed them.” In regard to standard issues, one of the experienced nurses stated that: “like other developed countries, as I was accountable for maximum 3-4 patients, you can expect me to do comprehensive care.” Contextual notes elucidated that this theme itself act as both a condition and consequence of reciprocal gap.

## Discussion

The findings of this study emphasized ambiguity in knowledge transfer and the reciprocal gap will continue to exist in this way of nursing in Iran. According to these results, we are in the starting point in the field of study on knowledge translation and utilization that are discussed in the scholar writings or published papers under title of research-based knowing and knowledge. As other research findings in Iran indicated, research utilization in clinical settings has been limited due to lack of management support, time inadequacy secondary to work overload, lack of skill and knowledge to do research, and consequently, establishment of knowledge-based practice depend on educational and organizational efforts and restructurings.^17^

Our findings within the theme of “clinical behavior structure” proposed that practical nurses and educators are incompetent in delivering a new version of nursing. Other studies in Iran confirmed that staff’s lack of knowledge and clinical educators’ lack of skills are major factors affecting competency and productivity of nurses.^18^,^19^,^21^ Moreover, causal condition of this process has been interrelated to inadequate essential professional competency and qualification in educational and transition period.^15^,^16^ Also, the clinical environment was not a suitable place to experience the integration between theory and practice. While, Alavi et al^27^ findings indicated that through making closed relationships with nursing staffs, students accept themselves as a component of caring team, so they can share and benefit from group synergies. As well, Hewison and Wildman^7^ claimed that the predominant values of practice are likely to dominate, again emphasizing the new dimension of the theory-practice gap. Furthermore, Chun-Heung and French^28^ stated that both nurse tutors and practitioners should have access to continuing education to enable them to take up a clinical teaching role as well as to maintain competency in clinical nursing practice; where, our findings within the theme of “paradoxical knowledge and practice” indicated that there is not a competent educational structure and content. The content of this recent theme implies that the students, their clinical teachers, and junior nurses acted as followers of existing routines in their clinical education, which in turn, would widen the gap and act against knowledge transfer. Greenwood^29^ alludes to the notion of desensitization of some student nurses during their professional socialization, and claims that the education of nurses often led to nurses compartmentalizing, in their minds, concepts of theory separate from concepts for practice. This results in “utilitarian nursing”, which in turn, is the outcome of a dichotomy between the ideal and real world.^10^ Research findings in Iran indicated that the culture of nursing in Iran is perceived as highly task oriented and physician-centered.^30^ Furthermore, Salsali et al^31^ found that there was a less caring attitude among nurses with more technical and mechanical skills without even any standard repertoire of procedures, thereby providing less individual and holistic care. Indeed, the practice settings were predominantly oriented towards task completion, and consequently such factors inhibited the effective clinical preparation towards the integration of theory and practice.

“The competency of clinical educator” besides “formless teaching-learning process” are two interrelated sub-themes of “paradoxical knowledge and practice”, as other important intervening conditions influencing educator ability to integrate the theoretical and practical knowledge. Corlett et al^13^ declared that “the nurse teacher did not have recent clinical experience of the specialties involved, but routinely taught theoretical and practical elements associated with the first year syllabus.” Farhadian et al^32^ concluded that the clinical educators needed to get more information and skills on clinical education to be able to integrate theory to practice. Also, comments from the participants included the requirement for encouraging critical thinking, problem solving, and teaching methods to foster a more realistic outlook of student and practice outcomes. The importance of this style has been well documented.^7^,^13^,^33^–^35^

The concept of “educational content inadequacy” within the theme of “paradoxical knowledge and practice” implies that nursing educational content for undergraduate nursing was perceived as medical-centered, noncontextual and non-nursing. Walsh and Jones^35^ discussed that the education must be contextspecific, i.e. specifically dedicated to educational packages for each area of practice that facilitates greater understanding and enhances clinical application and performance. As well, Prowse and Lyne^36^ declared that learning is effective when knowledge use is contextualized rather than abstract. Knight et al^33^ concluded that perhaps, by identifying the various facets of knowledge required to support nursing skills, the true value of nursing care will be established. Where, it has been concluded that Iranian nurses are still seen as mere handmaidens to the doctors or simply dutiful employees at a hospital, both in the public and professional contexts.^30^ In addition, Hoseini et al^37^ findings showed that from seventeen skills studied in their research, the following have not met the expectation of professors and graduated students: subcutaneous injection, blood transfusion, urinary catheterization, nasogastric tube insertion, lavage and enema with the average frequency of 0.06, 1.49, 0.79, 0.80, 0.08 and 0.17, respectively. Moattari et al^38^ findings demonstrated that the process of reflective thinking influencing clinical experience emerges in 5 domains of caring, thinking, theory and practice integration, self-regulatory mechanisms and motivation.

The concept of “unstructured evaluation” as another subtheme of “paradoxical knowledge and practice” implies that there is an unstructured evaluation system at both practice and education setting in order to make an objective evaluation decisions for students and staff. French et al^39^ in an international comparative study found that assessment methods in nursing were largely summative. While, Alavi et al^40^ findings emphasized that the multidimensional approaches should be adopted for comprehensive clinical evaluation. Also, Salsali^31^ stated faculty evaluation must be approached more analytically, objectively, and comprehensively to ensure that all nursing educators receive the fairest treatment possible and that the teachinglearning process is enhanced.

The concept of “divergence in nursing organization” includes interrelated concepts of “content dimensions” and “structural dimensions”, implies that nursing organization including service and education are completely divergent. As a whole picture of culture and environment in Iranian nursing, it is difficult to find a harmony between content and structural dimensions. Whilst et al^41^ found that there is a significant and positive relationship between total scores of nurses’ affective organizational commitment and work environment conditions. Furthermore, Yaghoubi et al^42^ elucidated that there was a significant relationship between learning organization and organizational commitment and there was a significant relationship between learning organization and job experience.

Moreover, another aspect of culture revealed that the culture of nursing was perceived as highly task-oriented and physician-centered. Adib et al^18^ confirmed these findings. As well, Gerrish and Griffith^43^ reported that all overseas nurses commented on differences in the organization of nursing care. Many were familiar with a more task-oriented approach to practice.

Based on “hierarchy of authority’ concept, there are several nursing policy-making authorities in service and education that are completely parallel in their activities. Therefore, due to this divergent, knowledge transference and correlation between service and education in order to integrate theory and practice has been reciprocally influenced. Adib-Hajbaghery^44^ found that factors such as unsupportive management, weak teamwork and ignorance on the part of senior management of the value of scientific work make the work environment inimical to the implementation of evidence-based nursing. In addition, Hewison and Wildman^7^ concluded that the future of nursing can be secured, but it will involve partnership and cooperation with the new managers. Regarding the concept of “hierarchy of authority”, one of the ways suggested by the participants was allocation of bilateral responsibilities between practice and education. Salvoni^6^ stated that the rational for these posts, i.e. “joint appointments” are: facilitating the application of theory to practice, promoting effective collaboration between service and education, promoting research based practice, and developing nursing practice.

The concepts of “standard” and “expertise” revealed that nurses wanted to have expert field working and standardization in the nursing system such as patient/nurse ratio, standard procedures, to do better within the nursing domain. Mirazabeigi et al^45^ indicated that educated master degree nurses have not necessary clinical competency, and accordingly, 92.7% of them supposed the specialization programs in nursing that would be resulted in quality care promotion, specialized field of work, appropriate job status, and decreased cost of delivered health care.

Additionally, Houser^46^ demonstrated that greater expertise can have a major influence on the outcome of patient care.

## Conclusion

In this paper we have shown the reciprocal relationship between service and education in order to reveal the “whats” and “hows” of nursing practice. Traditional routine-based paradigm is the dominant picture of Iranian nursing in practice and education at the baccalaureate level. Indeed, this indicated that nursing discipline in Iran is experiencing a theory-to-practice, and practice-to-theory gap that would move forward the knowledge translation ambiguously and put the discipline at risk. So, the big question you may ask is, *so what?*

An Iranian proverb says: “a scholar without practice is like a bee without honey”. And, “the shortest answer is doing” as an English prov-erb says.

The authors declare no conflict of interest in this study.

## References

[1] Castledine G (1996). Clarifying and defining nursing role developments. Br J Nurs.

[2] Salsali M (2000). The development of nursing education in Iran. Int Hist Nurs J.

[3] Cheraghi MA, Salsali M, Ahmadi F (2007). Iranian nurses’ perceptions of theoretical knowledge transfer into clinical practice: A grounded theory approach. Nursing and Health Sciences.

[4] Cheraghi MA, Salasli M, Ahmadi F (2008). Factors influencing the clinical preparation of BS nursing student interns in Iran. Int J Nurs Pract.

[5] Salsali M (2005). Evaluating teaching effectiveness in nursing education: an Iranian perspective. BMC Med Educ.

[6] Salvoni M (2001). Joint appointments: another dimension to building bridges. Nurse Educ Today.

[7] Hewison A, Wildman S (1996). The theory-practice gap in nursing: a new dimension. J Adv Nurs.

[8] Severinsson EI (1998). Bridging the gap between theory and practice: a supervision programme for nursing students. J Adv Nurs.

[9] Gerrish K (2000). Still fumbling along? A comparative study of the newly qualified nurse’s perception of the transition from student to qualified nurse. J Adv Nurs.

[10] Henderson S (2002). Factors impacting on nurses’ transference of theoretical knowledge of holistic care into clinical practice. Nurse Education in Practice.

[11] Rolfe G (1996). Closing the theory-practice gap: a new paradigm for nursing.

[12] Corlett J (2000). The perceptions of nurse teachers, student nurses and preceptors of the theory-practice gap in nurse educa-tion. Nurse Educ Today.

[13] Corlett J, Palfreyman JW, Staines HJ, Marr H (2003). Factors influencing theoretical knowledge and practical skill acquisition in student nurses: an empirical experiment. Nurse Educ Today.

[14] Maben J, Latter S, Clark JM (2006). The theory-practice gap: impact of professional-bureaucratic work conflict on newly-qualified nurses. J Adv Nurs.

[15] Abedi HA, Heidari A, Salsali M (2004). Nursing graduates’ experience of professional prepared during transition to their clinical role. Iranian Journal of Medical Education.

[16] Valizadeh S, Abedi HA, Zamanzadeh V, Fathi Azar E (2007). Challenges of nursing students during their study, A qualitative study. Iranian Journal of Medical Education.

[17] Mehrdad N, Salsali M (2009). Iranian nurses’ constraints for research utilization in clinical setting. Iran Journal of Nursing.

[18] Adib HM, Salsali M, Ahmadi F (2004). A qualitative study of Iranian nurses’ understanding and experiences of professional power. Hum Resour Health.

[19] Nayeri ND, Nazari AA, Salsali M, Ahmadi F (2005). Iranian staff nurses’ views of their productivity and human resource factors improving and impeding it: a qualitative study. Hum Resour Health.

[20] Hagbaghery MA, Salsali M, Ahmadi F (2004). The factors facilitating and inhibiting effective clinical decision-making in nursing: a qualitative study. BMC Nurs.

[21] Sharif F, Masoumi S (2005). A qualitative study of nursing student experiences of clinical practice. BMC Nurs.

[22] Strauss AL, Corbin JM (1998). Basics of qualitative research: techniques and procedures for developing grounded theory.

[23] Strauss AL, Corbin JM, Denzin MK, Lincoln YS (2003). Grounded theory methodology. Strategies of qualitative inquiry.

[24] Denzin NK, Lincoln YS (2005). The SAGE handbook of qualitative research.

[25] Strauss AL, Corbin JM (1990). Basics of qualitative research.

[26] Speziale HS, Carpenter DR (2003). Qualitative research in nursing: advancing the humanistic imperative.

[27] Alavi M, Tavakol K, Behzad nejad M, Mahdi Zadeh K (2008). Iranian nursing students’ experiences of relationships with nurses in clinical setting. IJNMR.

[28] Chun-Heung L, French P (1997). Education in the practicum: a study of the ward learning climate in Hong Kong. J Adv Nurs.

[29] Greenwood J (1993). The apparent desensitization of student nurses during their professional socialization: a cognitive perspective. J Adv Nurs.

[30] Nikbakht Nasrabadi A, Emami A, Parsa Yekta Z (2003). Nursing experience in Iran. Int J Nurs Pract.

[31] Salsali M, Cheraghi MA, Ahmadi F (2009). Organizational factors influencing knowledge transfer into practice in Iranian nursing context: A grounded theory approach. Int J Nurs Pract.

[32] Farhadian F, Totoonchi M, Changiz T, Haghani F, Oveise-Gharan S (2007). Faculty members’ educational needs and skills of Isfahan University of Medical Sciences on clinical teaching approaches. Iranian Journal of Medical Education.

[33] Knight CM, Moule P, Desbottes Z (2000). The grid that bridges the gap. Nurse Educ Today.

[34] Lambert V, Glacken M (2004). Clinical support roles: a review of the literature. Nurse Educ Pract.

[35] Walsh P, Jones K (2005). An exploration of tripartite collaboration in developing a strategic approach to the facilitation of practice learning. Nurse Educ Pract.

[36] Prowse MA, Lyne PA (2000). Clinical effectiveness in the post-anaesthesia care unit: how nursing knowledge contributes to achieving intended patient outcomes. J Adv Nurs.

[37] Hoseini SA, Islamian J, Bakhtiari S (2009). Basic clinical skills of nursing students: a comparison between nursing students, nursing graduates‘ and lecturers’ viewpoints. IJNMR.

[38] Moattari M, Abedi HA (2008). Nursing students’ experience on reflection. Iranian Journal of Medical Education.

[39] French P, Anderson J, Burnard P, Holmes C, Mashaba G, Wong T (1996). International comparison of baccalaureate nursing degrees: collaboration in qualitative analysis. J Adv Nurs.

[40] Alavi M, Irajpour AR, Abedi HA (2007). Some concepts in the evaluation of clinical education: a qualitative study on the experiences of nursing students and clinical teachers. Strides in development of Medical Education.

[41] Vanaki Z, Vagharseyyedin SA (2009). Organizational commitment, work environment conditions, and life satisfaction among Iranian nurses. Nurs Health Sci.

[42] Yaghoubi M, Raeisi AR, Afshar M, Yarmohammadian MH, Hasanzadeh A, Javadi M (2010). The relationship between the learning organization and organizational commitment among nursing managers in educational hospitals of Isfahan University of Medical Sciences in 2008-9. IJNMR.

[43] Gerrish K, Griffith V (2004). Integration of overseas Registered Nurses: evaluation of an adaptation programme. J Adv Nurs.

[44] Adib-Hajbaghery M (2007). Factors facilitating and inhibiting evidence-based nursing in Iran. J Adv Nurs.

[45] Mirzabeigi GH, Sanjari M, Salemi S, Babaei F, Kheradmand M (2009). Iranian nursing and midwifery faculties’ perspectives on necessity of specialized programs in master degree. Iranian Journal of Medical Education.

[46] Houser J (2003). A model for evaluating the context of nursing care delivery. J Nurs Adm.

